# VPO1 Mediates ApoE Oxidation and Impairs the Clearance of Plasma Lipids

**DOI:** 10.1371/journal.pone.0057571

**Published:** 2013-02-25

**Authors:** Youfeng Yang, Zehong Cao, Ling Tian, W. Timothy Garvey, Guangjie Cheng

**Affiliations:** 1 Division of Pulmonary, Allergy and Critical Care Medicine, Department of Medicine, University of Alabama at Birmingham, Birmingham, Alabama, United States of America; 2 Department of Nutrition Sciences, University of Alabama at Birmingham, Birmingham, Alabama, United States of America; 3 The Birmingham VA Medical Center, Birmingham, Alabama, United States of America; Nihon University School of Medicine, Japan

## Abstract

**Objective:**

ApoE is an abundant component of chylomicron, VLDL, IDL, and HDL. It binds to multiple types of lipids and is implicated in cholesterol and triglyceride homeostasis. Oxidation of ApoE plays a crucial role in the genesis of atherosclerosis. It is proposed that heme-containing peroxidases (hPx) are major mediators of lipoprotein oxidization. Vascular peroxidase 1 (VPO1) is a recently-discovered hPx, which is expressed in cardiovascular system, lung, liver etc. and secreted into plasma. Its plasma concentration is three orders of magnitude of that of myeloperoxidase. If VPO1 mediates ApoE oxidation and affects the lipid metabolism remains to be elucidated.

**Methods:**

Recombinant ApoE and VPO1 were expressed and purified from stably-expressing cell lines deriving from HEK293 cells. ApoE oxidation was carried out by VPO1 in the presence of H_2_O_2_ and chloride. ApoE oxidation was verified by a variety of approaches including immunoblot and amino acid analyses. To evaluate the functional changes in VPO1-oxidized ApoE, lipid emulsion particle binding assays were employed.

**Results:**

Oxidized ApoE binds weaker to lipid emulsion particles, which mimic the large lipid complexes *in vivo*. In lipid efflux assay, oxidized ApoE showed reduced capability in efflux of lipids from foam cells. Mice administrated with oxidized ApoE *via* blood exhibited weaker clearance ability of plasma lipids.

**Conclusions:**

Our data suggest that VPO1 is a new mediator regulating lipid homeostasis, implying a role in genesis and development of atherosclerosis.

## Introduction

The physiological functions of lipoproteins are to transport lipids in blood and facilitate lipids through the cellular membrane into cells. Liver, intestine, and adipose tissue are the major tissues for metabolism and storage of lipids [1]. Blood lipoproteins form five types of lipoprotein particles based on electrophoresis and ultracentrifugation, named high density lipoprotein (HDL), low density lipoprotein (LDL), intermediate-density lipoprotein (IDL), very low density lipoprotein (VLDL), and chylomicrons [2]. These particles have distinct functions in homeostasis and transportation of lipids.

Apolipoprotein E (ApoE) is a glycoprotein of 299 amino acids with a molecular mass of ∼34 kDa. It is a major component of chylomicron, VLDL, IDL and HDL [3], [4]. It is expressed in multiple tissues including liver, brain, kidneys, and spleen, and secreted into plasma. The major function of ApoE is proposed to facilitate lipid transportation [5]. Pathologically, ApoE plays similar roles in genesis of atherosclerosis in both mouse and humans. ApoE-deficient mice showed severe hypercholesterolemia and advanced atherosclerotic lesion [6], [7]. In this animal model, formation of foam cells and development of fibroproliferative lesions are histochemically similar to that of human atherosclerosis [8], [9]. Formation of foam cells is the benchmark of atherosclerosis. On the other hand, elevated plasma ApoE levels reduced hyperlipidemia and atherosclerosis such as in the transgenic mouse models with macrophage-specific ApoE expression. ApoE-deficient mice transplanted with bone marrow cells containing human ApoE gene revealed elevated plasma ApoE levels, decreased blood lipid levels, and less atherosclerotic lesions [10], [11], [12].

Atherosclerosis is a metabolic and inflammatory disorder which characterized by reduction in the luminal diameter of the main arteries [13]. The first stage in the development of atherosclerosis is the formation of foam cells on the wall of blood vessel [14]. Foam cells derived from blood monocytes by the uptake and accumulation of lipids, mainly cholesterol and triglycerides, resulting in impaired transportation of lipids. It is assumed that the impaired transportation and accumulation of lipids are caused by the modification and dysfunction of lipoproteins, e.g. ApoE. A line of evidence shows that animal heme-containing peroxidases (hPxes) play an important role in oxidation of lipids and proteins, and development of atherosclerosis [15]. Myeloperoxidase (MPO) is proto-enzyme of the family and is extensively studied. MPO is restricted expressed in neutrophils and monocytes. Only a small fraction of MPO is secreted into plasma by a process called “leaking”. It is proposed that monocytes migrate into the intermediate of the vessel walls at inflammatory sites. Macrophages, which are derived from monocytes, then oxidize LDL and form foam cells [16]. MPO mediates the oxidation of proteins, lipids and DNA by generating potent hypochlorous acid (HOCl), a key mediator in the genesis of atherosclerosis [16], [17].

Recently, our group identified and characterized a novel hPx, named vascular peroxidase 1 (VPO1) [18]. It is mainly expressed in the cardiovascular system, lung, liver, and spleen, and most of VPO1 proteins are secreted into blood stream with the concentration of three orders of magnitude of that of MPO in plasma [19]. Like MPO, VPO1 is able to oxidize chloride to generate HOCl in the presence of hydrogen peroxide (H_2_O_2_) [20], [21]. However, the pathophysiological role of VPO1 in genesis and development of atherosclerosis remains unclear. In the present study, we showed that ApoE and VPO1 are localized in the same area of atherosclerotic plaques. ApoE is susceptible to oxidation by VPO1. Moreover, oxidized ApoE (ox-ApoE) has decreased functions in promoting triglyceride and cholesterol efflux from foam cells, and in plasma lipid clearance. These data suggest that VPO1 participates in the dysfunction of ApoE and the formation of atherosclerosis.

## Materials and Methods

### Ethics Statement

The experiments on mice were approved by the University of Alabama at Birmingham Animal Care and Use Committee (Animal Project Number: 120309582).

### Cells and Reagents

HEK293H (Invitrogen, Carlsbad, CA) cells were maintained in DMEM media with 10% fetal bovine serum, 100 U/ml penicillin, and 100 µg/ml streptomycin. THP-1 cells (human acute monocytic leukemia cell line, American Type Culture Collection, Manassas, VA) were maintained in RPMI 1640 media with 10% fetal bovine serum, 100 U/ml penicillin, and 100 µg/ml streptomycin. Trinitrobenzene sulfonic acid, sodium hypochlorite solution, triolein, egg phosphatidylcholine (PC), PC assay kit, and NaCl were purchased from Sigma–Aldrich (St. Louis, MO); chemiluminescent substrate for immunoblots was from Pierce Biotechnology (Rockford, IL); goat polyclonal antibody against human ApoE was from Millipore (Billerica, MA); cholesterol assay kit was from Wako Chemicals (Richmond, VA); triglycerides assay kit was from Pointe Scientific (Lincoln Park, MI); Fluorescence-labeled second antibodies were from Invitrogen (Carlsbad, CA). Recombinant VPO1 proteins were expressed and purified as described in the article [18].

### Mice

C57BL/6J mice were originally obtained from the Jackson Laboratory and maintained in micro-isolator cages. The experiments on mice were carried out according to the guidelines of the University of Alabama at Birmingham Animal Care and Use Committee. Unless otherwise stated, mice were fed with normal chow diet. Mice at 8–10 weeks were used in the experiments.

### Immunofluorescence Assay

Cryosections of atherosclerotic aorta from low-density lipoprotein receptor (LDLR)−deficient mice (generously provided by Dr. Janusz H. Kabarowski at Department of Microbiology, University of Alabama at Birmingham) were fixed by incubation in cold acetone (−20°C) for 2 minutes and rehydrated in phosphate buffered saline (PBS). Standard immunofluorescence staining was carried out. The primary antibodies were polyclonal goat anti-ApoE antibody (Millipore, Billerica, MA) and affinity-purified rabbit anti-VPO1 antibody as described in the article [18]. The secondary antibodies were Alexa® Fluor 488-conjugated anti-goat antibody and Alexa® Fluor 555-conjugated anti-rabbit antibody. Images were taken and analyzed by Zeiss Axiovert fluorescence microscope (Carl Zeiss, Heidelberg, Germany).

### Establish ApoE3-stably Expressing Cell Lines

Full-length human ApoE3 gene was constructed in plasmid pcDNA3. The plasmid containing ApoE3 was transfected into HEK293 cells using FuGENE® 6 (Roche, Indianapolis, IN) following the manufacturer’s instruction. After 48 hrs, cells were re-plated and G418 was added into medium at 500 µg/ml. Media containing G418 were changed every 3 days for 3 weeks. The positive colonies of ApoE3-stably expressing cell lines were screened by immunoblot analysis using anti-ApoE antibody.

### Expression and Purification of ApoE3

For expression and purification of recombinant ApoE3 (rApoE), ApoE3-stably expressing cells were cultured in spinner bottle in serum-free medium [DMEM medium supplemented with 0.1% (w/v) Pluronic® F68 and 3.6 g/L CDM-HD (FiberCell Systems Inc. Frederic, MD)]. In brief, ∼0.5×10^6^ cells/ml were grown in 100 ml spinner bottles with 40 ml of the serum-free medium at 37°C and 8% CO_2_ atmosphere with stirring at 100 rpm. After three days, 60 ml of fresh serum-free medium was added and the culture was continued for four days. Subsequently, the culture was transferred to a 1000 ml spinner bottle and 400 ml fresh media was added. When cell density reached 3×10^6^/ml, 400 ml fresh media was added and culture was continued for another 3–4 days. The supernatant was harvested by centrifugation at 1000×*g* at 4°C for 10 min. The conventional procedures of chromatography were carried out for purification of rApoE. The supernatant was loading into a Q-Sepharose column (1×8 cm) with speed at 0.5 ml/min. The column was washed with 100 ml of 20 mM phosphate buffer, pH8.0, and gradually eluted by 60 ml of 20 mM phosphate buffer containing 0–0.6 M NaCl. rApoE was detected by SDS-PAGE following Coomassie Blue staining and immunoblot analysis. The elution containing rApoE and ∼0.4 M NaCl was subject to be concentrated by using GE Vivaspin® 20 (10 kDa MWCO) (GE Healthcare Biosciences, Pittsburgh, PA). The concentrated sample was loaded onto a Sephacryl S-300 column (1×100 cm) for further purification. The column was eluted with phosphate buffer at 0.3 ml/min and samples were collected at 4 ml/tube. A_280_ was monitored. The purity of rApoE was determined by SDS-PAGE. Single band was observed. Protein concentration was measured by Bradford dye-binding procedure using protein assay kit (Bio-Rad, Hercules, CA).

### Preparation of Lipoproteins

VLDL was isolated from human plasma by ultracentrifugation as described previously [22]. Briefly, VLDL was isolated by ultracentrifugation at plasma density (d = 1.006 g/cm^3^), at 100,000×g for 18 hrs at 12°C. VLDL was dialyzed against 20 mM phosphate buffer, pH 7.4, before use.

### Oxidation of rApoE and VLDL

rApoE or VLDL was incubated in 800 µM HOCl, or in the presence of 50 µM H_2_O_2_, 140 mM NaCl, 750 nM/heme VPO1 or 50 nM/heme MPO at 37°C for 24 hrs. The reactions were terminated by dialysis in 20 mM phosphate buffer, pH 7.4.

### Western Blot Analysis

Protein samples were resolved by 12% SDS-PAGE and transferred to polyvinylidene difluoride (PVDF) membranes using a Mini-Tank Transfer System (Bio-Rad Laboratories, Hercules, CA). Proteins were detected by chemiluminescence (Pierce, SuperSignal West Pico Chemiluminescent Substrate) with the primary antibody as indicated.

### Measurement of Free Amino Groups

Free Amino acid groups of apolipoprotein were quantified as describe previously [23]. Briefly, samples were mixed with 1 ml of 4% NaHCO_3_ (w/v; pH 8.4) and 50 µl 0.1% (v/v). trinitrobenzene sulfonic acid. After incubation at 37°C for 1 hr, 100 µl of 1 M HCl and 100 µl of 10% SDS were added. Absorbance at 340 nm was recorded.

### Measurement of Tryptophan Residues

Content of tryptophan residues was evaluated as described previously [24]. Briefly, native ApoE or ox-ApoE was measured by fluorescence (emission 335 nm/excitation 280 nm) with a BioTek Microplate Reader (Winooski, VT). Fluorescence intensity was normalized with ApoE protein concentration.

### Preparation of Emulsion Particles

Emulsion particles were prepared by using sonication and ultracentrifugation as described in [25]. In brief, triolein and egg PC emulsion particles were prepared by mixing triolein and PC with the weight ratio of 3.5∶1 while PC emulsion particles contained only PC. To prepare triolein and egg PC emulsion particles, 5 µl of 0.1 mg/µl egg PC and 1.75 µl of 1 mg/µl triolein in chloroform/methanol solution were mixed in a glass tube and dried under a stream of nitrogen. The tube containing triolein and egg PC was placed into a vacuum desiccator overnight. The dry lipids were suspended in Tris buffer (10 mM Tris-HCl, pH 7.4, 150 mM NaCl, 0.02% NaN_3_, 1 mM EDTA) and sonicated for 30 min at 50–60°C under a stream of nitrogen. Titanium was removed from the crude emulsions by centrifuging at 3000 rpm for 15 min. The samples were then centrifuged at 15,000 rmp for 15 min at 20°C to remove creamy layer and washed twice with the buffer. Additional spinning at 17,000 rpm for 1 hr at 20°C min to remove any contaminated liposome. The resulting creamy top layer was collected as emulsion particles. After purification, the final PC concentration in solution is ∼0.3 mg/ml while triolein is ∼1 mg/ml.

### Binding of ApoE or ox-ApoE to Emulsion Particles

Binding of ApoE or ox-ApoE to emulsion particles was assayed with a centrifugation method as described in [25]. The emulsion particles were incubated with ApoE or ox-ApoE for 1 hr at room temperature with gentle shaking in Tris buffer (pH 7.4) containing 0.25 M sucrose. The final concentrations of ApoE, PC and triolein were 20 µg/ml, ∼0.03 mg/ml and ∼0.1 mg/ml, respectively. After incubation, the samples were centrifuged in a Beckman ultracentrifuge with a 50 Ti rotor at 30,000 rpm for 30 min. All of lipids were found in the top fraction and free ApoE was in the down layer. The top and lower layers were collected. Equal amount of samples from top or lower layers were carried out for Western blot analysis. The ratio was calculated as emulsion particle-bound ApoE/free ApoE.

**Triglyceride, cholesterol and PC assay** To load lipids, THP-1 cells were treated with 100 nM phorbol myristate acetate for 24 hrs, rested another 24 hrs, and then incubated with acetylated-LDL (Ac-LDL) for 24 hrs. LDL enriched cells were washed with PBS and incubated with medium containing 50 mg/L native or ox-ApoE. After 24 hrs, cells were extracted using lysis buffer (CelLytic M, Sigma-Aldrich St. Louis, MO). The cellular triglycerides, cholesterol and PC were measured by Pointe Scientific triglycerides assay kit, Wako cholesterol assay kit, and Sigma–Aldrich PC assay kit, respectively, following the manufacturers’ instructions. For detection lipids in supernatant, LDL-enriched cells were washed with PBS and incubated with medium containing 100 mg/L native or ox-ApoE. After 9 hrs, triglycerides, cholesterol and PC in supernatant were measured by the relative kit. As foam cells are full of engulfed lipid vesicles, the levels of free glycerol/glycerol-3-phosphate are negligible as our previous report [26].

### Assays for Mouse Plasma Triglyceride and Cholesterol Concentrations

C57BL/6J mice were deprived of food for 16 hrs before the administration of native or ox-ApoE proteins. Blood volume of mouse is calculate by 58.5 ml per kilogram mice body weight [27], and the endogenous ApoE concentration in mouse serum is 38.7 mg/L [27]. The final concentration of administered rApoE is 60 mg/L. Venous blood was drawn for analysis before and after 1 hr of the injection. Mouse plasma was collected immediately and the triglyceride and cholesterol concentrations were measured as described above. This study was carried out in strict accordance with the recommendations in the Guide for the Care and Use of Laboratory Animals of the National Institutes of Health. The protocol was approved by the Institutional Animal Care and Use Committee of the University of Alabama at Birmingham (Animal Project Number: 120309582).

### Statistical Analysis

Data are shown as means ± SEM, unless otherwise indicated. Quantitative variables were compared by means of ANOVA followed by Tukey's multiple comparison tests. A value of P<0.05 was considered significant.

## Results

### VPO1 and ApoE are Localized at Atherosclerotic Lesions

**Figure 1:**
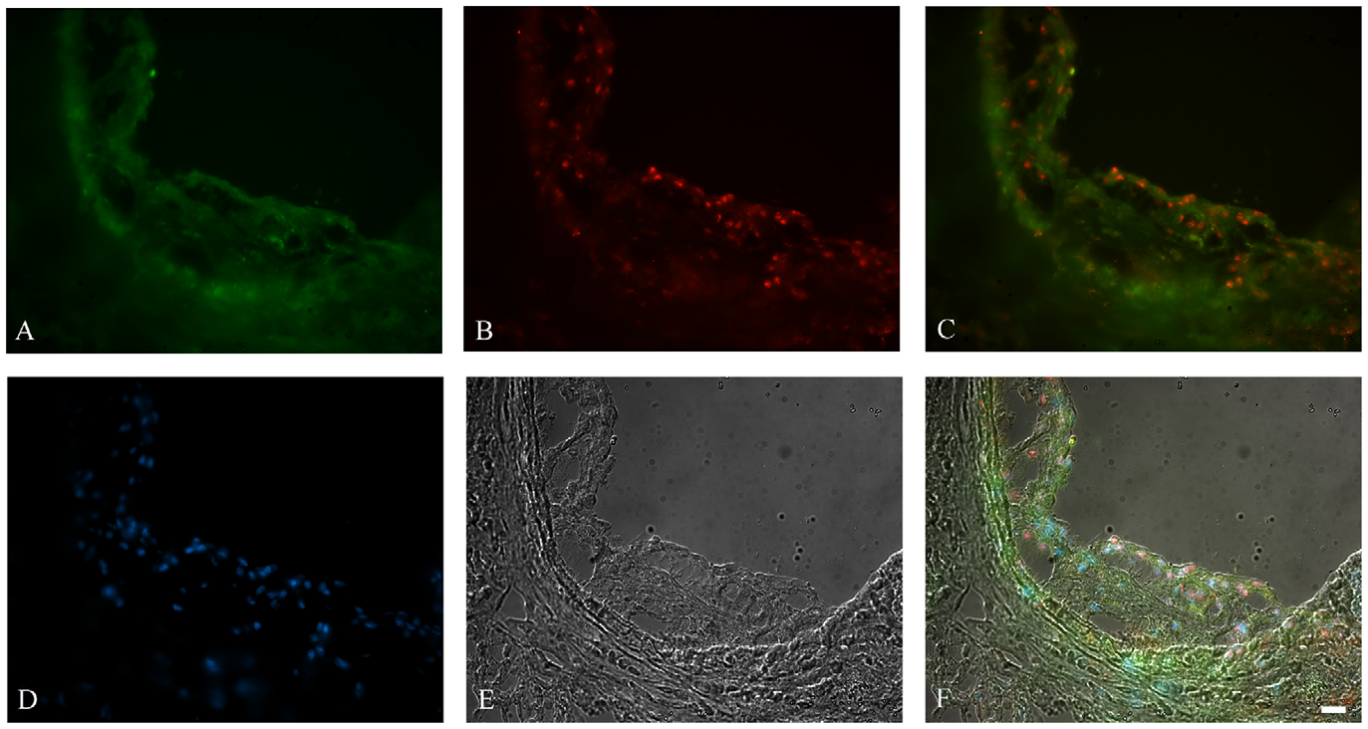
Localization of VPO1 and ApoE in atherosclerotic lesion. Immunofluorescence staining was carried out for detection of ApoE (A) and VPO1 (B) using sections from mouse atherosclerotic lesions. C. Merged image of A and B. D. Nuclei were visualized with Hoechst staining (blue). E. Bright-field image. F. Merged image of A, B, D, and E. Scale bar, 20 µm. Magnification: x400.

Previous reports demonstrated that ApoE localized at lipid streaks and atherosclerotic plaques [28], [29]. Our immunofluorescence assay confirmed the specific localization of ApoE in aortic atherosclerotic plaques using LDLR-deficient mice (Fig. 1A). VPO1 is new hPx expressed in vascular endothelial cells and small muscle cells. We evaluated if VPO1 is co-localized with ApoE in atherosclerotic plaques. Immunofluorescence assay showed that VPO1 was also located in the atherosclerotic plaque and was close to ApoE (Fig. 1). H_2_O_2_ and chloride are the co-substrate of hPxes and virtually, all cells in vessel walls produce H_2_O_2_ in varying amounts and in response to diverse stimuli [30] while chloride readily exists in cells with physiological concentration of 100–140 mM. Thus, our data support that VPO1 is proximal to ApoE and potentially mediates oxidation of ApoE.

### Oxidation of ApoE by VPO1/H_2_O_2_/Cl^−^ System

**Figure 2:**
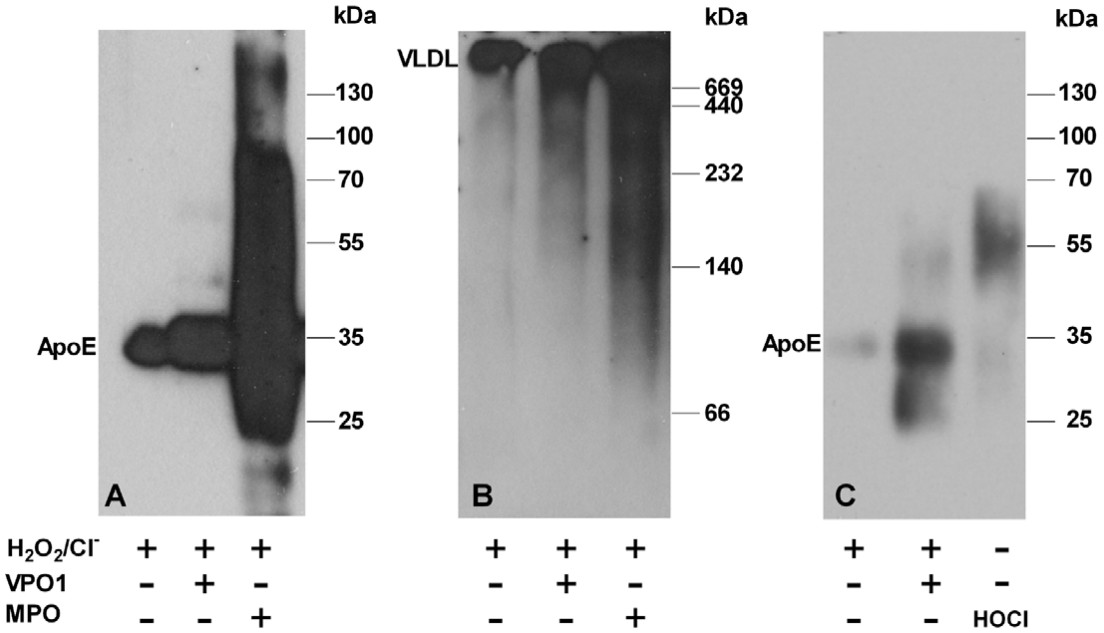
Immunoblot analysis of ApoE oxidized by VPO1. A. VLDL was oxidized by MPO or VPO1 as described in Materials and Methods. The oxidized lipoproteins were separated by SDS-PAGE and transferred to PVDF membrane. The blot was analyzed by using anti-ApoE antibody and visualized using chemiluminescence. B. The same as in A, but the samples were separated by using native PAGE. C. rApoE was oxidized by VPO1 or reagent HOCl. Blot was analyzed as in A.

An array of evidence shows that ApoE is highly susceptible to oxidation by MPO [31]. Treatment of VLDL with MPO lead to a variety of oxidations of ApoE proteins including the formation of intra- and intermolecular crosslinks with the concomitant formation of high molecular mass aggregates [32], [33]. The peroxidase domain of VPO1 is highly similar to MPO and is able to generate HOCl in the presence of H_2_O_2_ [18], [20], [21]. To evaluate the oxidation of ApoE by VPO1, we incubated VLDL or rApoE with VPO1/H_2_O_2_/Cl^−^, comparing with MPO/H_2_O_2_/Cl^−^ system. Oxidation of ApoE was examined by immunoblot analysis. The membranes were blotted by anti-ApoE antibody. It is expected that the migration rate of ox-ApoE is different with the native ApoE. Indeed, ApoE in VLDL oxidized by VPO1/H_2_O_2_/Cl^−^ as well as by MPO/H_2_O_2_/Cl^−^ revealed distinct migration rates comparing with native ApoE controls. In addition to the 34 kDa band, which represents the native ApoE, the immunoreactive bands of ox-ApoE in VLDL showed additional molecular masses of 42 and 80 kDa (Fig. 2A). Some degraded bands in ox-ApoE were also observed (Fig. 2A). Similarly, rApoE oxidized by VPO1/H_2_O_2_/Cl^−^ or by MPO/H_2_O_2_/Cl^−^ revealed the patterns of immunoreactive bands as ox-ApoE in VLDL (Fig. 2A and 2C). Omitting H_2_O_2_ in the system, migration rates of rApoE did not change (Data not shown).

The oxidized VLDL was also separated using native PAGE and transferred to PVDF membrane for immunoblot analysis using anti-ApoE antibody (Fig. 2B). VLDL, which size is approximately 25–90 nanometers, stayed on top of the gel whereas the oxidized VLDL revealed smear bands below the main band in native VLDL (Fig. 2B). Taken together, our data suggest both ApoE in VLDL and rApoE are readily oxidized by VPO1/H_2_O_2_/Cl^−^, resulting in a variety of oxidations including intra- and intermolecular crosslinks, degradation etc.

### VPO1-mediated Oxidation of Amino Acid Residues in ApoE

**Figure 3:**
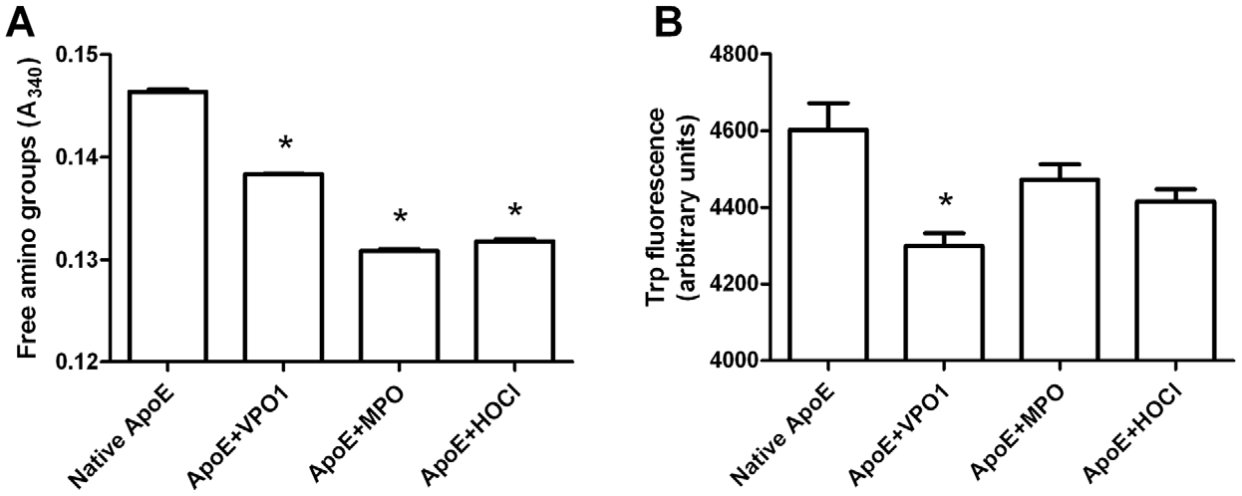
Loss of free amino groups and tryptophan residues in oxidized ApoE. A. Loss of free amino groups in oxidized ApoE. ApoE was oxidized by VPO1/H_2_O_2_/Cl^−^, MPO/H_2_O_2_/Cl^−^, or HOCl for 3 hrs at 37°C (n = 3). Unmodified amino groups were quantified with the method using trinitrobenzene sulfonic acid as described in Materials and Methods. Absorbance was measured at 340 nm. B. Loss of tryptophan residues following ApoE oxidation. ApoE was incubated with VPO1/H_2_O_2_/Cl^−^, MPO/H_2_O_2_/Cl^−^ or HOCl for 3 hrs at 37°C (n = 3) as in A. Fluorescence from free tryptophan residue (emission 335 nm/excitation 280 nm) was quantified with a BioTek Microplate Reader. Trp: tryptophan. Data are shown as means ± SEM; *p<0.05 *vs*. native ApoE; n = 3.

MPO/H_2_O_2_/Cl^−^ system generates potent oxidant, HOCl, and mediates oxidation of a variety of biomolecules including proteins, lipids and DNA. In atherosclerosis, lipoproteins are the common targets. Treatment of VLDL with reagent HOCl or MPO/H_2_O_2_/Cl^−^ resulted in the modification of the protein moiety and impairment of protein functions [34]. Lysine-derived amino groups are highly susceptible to HOCl-mediated attack [35]. We incubated rApoE with VPO1/H_2_O_2_/Cl^−^ and examined the status of its amino acid residues. Like MPO/H_2_O_2_/Cl^−^ systems or reagent HOCl, VPO1/H_2_O_2_/Cl^−^ mediated the loss of free amino groups (Fig. 3A). VPO1 showed weaker effects than MPO thought VPO1 concentration was 10 folds (500 nM) than that of MPO (Fig. 3A), consistent with our previous report that VPO1 activity was approximately one twentieth of that of MPO [18]. However, VPO1 is expressed in vessel walls and its concentration in plasma is 1000 folds than that of MPO [18], [19]. It is assumed that the ability of VPO1-mediated oxidation of free amino acids is higher than that of MPO in vessel walls. HOCl at (800 µM) showed a similar ability of oxidation as MPO at 50 nM. Tryptophan residues are also susceptible to oxidation by HOCl [36]. After incubation with VPO1/H_2_O_2_/Cl^−^, MPO/H_2_O_2_/Cl^−^ or reagent HOCl, the quantity of tryptophan residues in ApoE was measured by fluorescence assay (emission 335 nm/excitation 280 nm). We found approximately 6% of tryptophan residues was oxidized by VPO1 (p<0.05 *vs*. native ApoE) (Fig. 3B) similar to that by MPO and HOCl. The data suggest that multiple types of amino acid residues may be oxidized by VPO1/H_2_O_2_/Cl^−^ system like MPO/H_2_O_2_/Cl^−^ system and reagent HOCl.

### Ox-ApoE Binds Less Lipids in Emulsion Particles than Native ApoE

**Figure 4:**
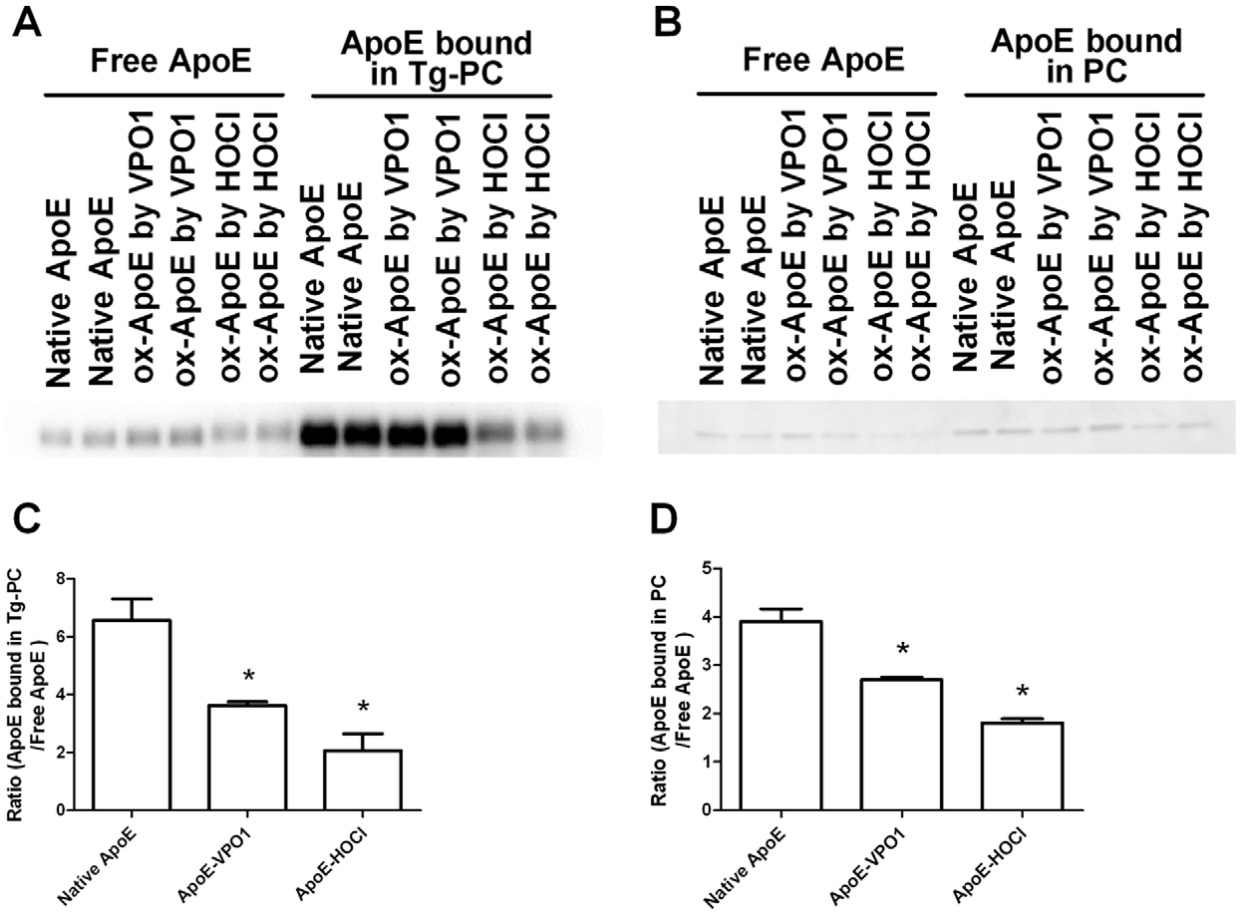
Ox-ApoE binds weakly to emulsion particles. A. Ox-ApoE binding with triglyceride-PC emulsion particles. Native and ox-ApoE were incubated with triglyceride-PC emulsion particles at room temperature for 1 hr with gentle shaking. Emulsion particles and lipid-free buffer were segregated by centrifugation. Equal amount of samples from top and lower layers (contain lipid-bound ApoE and free ApoE, respectively) were carried out for Western blot analysis using anti-ApoE antibody. B. Ox-ApoE binding with PC emulsion particles. Native and ox-ApoE were incubated with PC emulsion particles at room temperature for 1 hr with gentle shaking. Emulsion particles and lipid-free buffer were segregated by centrifugation as in A. Equal amount of samples from top and lower layers (contain lipid-bound ApoE and free ApoE, respectively) were carried out for Western blot analysis using anti-ApoE antibody. C. Quantification of ApoE and ox-ApoE binding to triglycerides-PC emulsion particles from data in A. The ratio was calculated as emulsion particle-bound ApoE/free ApoE. D. Quantification of ApoE and ox-ApoE binding to PC emulsion particles from data in B. The ratio was calculated as emulsion particle-bound ApoE/free ApoE. Data are shown as means ± SEM; *p<0.05. n = 3.

ApoE plays multiple roles in regulation of metabolism and transportation of lipids. Lipid molecules in the circulation system lie in two ways: as free lipids in very low concentration or in emulsion particles, such as the lipoprotein complexes VLDL, LDL, and HDL [21]. Most lipids in plasma exist in the lipoprotein complexes for facilitating transportation. Takahashi et al reported that rApoE enhanced the binding and internalization of triglyceride-rich lipoproteins to the VLDL receptor [37]. To determine the binding ability of ox-ApoE, emulsion particles from PC or Triolein-PC mixture were prepared by sonication and ultracentrifugation [25]. Emulsion particles were then incubated with ApoE or ox-ApoE. Emulsion particle-bound ApoE was then separated from unbound ApoE (free ApoE) by ultracentrifugation. The separated ApoE proteins were analyzed by immunoblot and quantified by using Image J software (The National Institutes of Health, Bethesda, MD). As shown in Fig. 4, ox-ApoE bound significantly less PC or Triolein-PC in emulsion particles than native ApoE.

### Ox-ApoE Shows Less Efflux of Triglycerides, Cholesterol, and PC from Foam Cells

**Figure 5:**
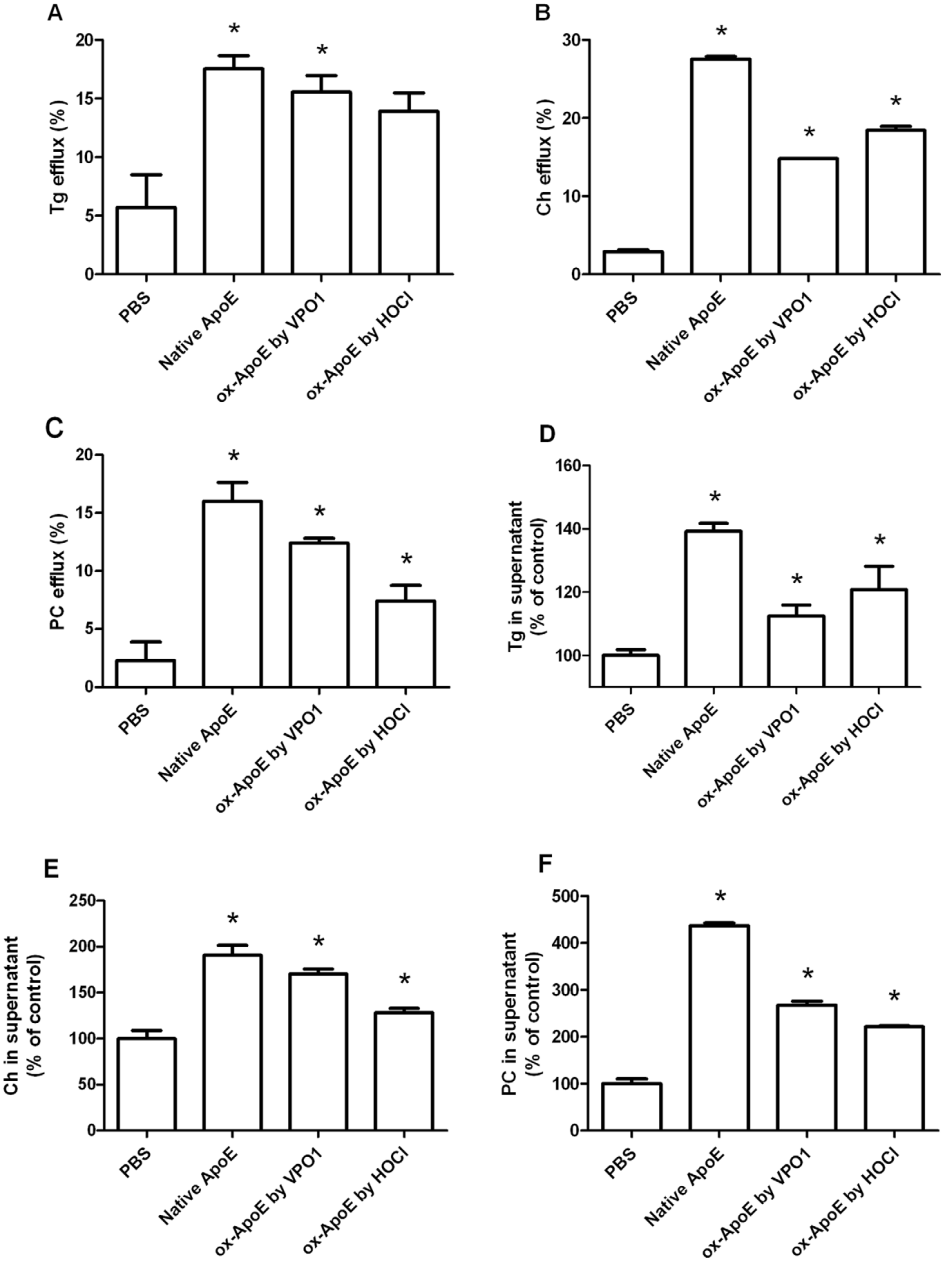
Effect of ox-ApoE on triglyceride, cholesterol and PC efflux from foam cells. A. Cellular triglyceride efflux by native and ox-ApoE. B. Cellular cholesterol efflux by native and ox-ApoE. C. Cellular PC efflux by native and ox-ApoE. D. Detection of triglycerides in supernatant. E. Detection of cholesterol in supernatant. F. Detection of PC in supernatant. THP-1 cells were loaded with lipids as described in Materials and Methods. Efflux of triglycerides, cholesterol, and PC was initiated by addition of native or ox-ApoE in cultured foam cells. After treatment of 9 hrs, triglycerides, cholesterol, and PC in supernatant were measured by the respect kit. After treatment of 24 hrs, the cellular triglycerides, cholesterol, and PC were measured with the respect kit. Tg: triglycerides; Ch: cholesterol; PC: phosphatidylcholine. Data are shown as means ± SEM; *p<0.05 vs. PBS group. n = 3.

It is reported that ApoE increased free cholesterol efflux into the medium [38]. ApoE as an extracellular receptor promoted cholesterol efflux activity at a dose-dependent manner [39]. To investigate the effect of rApoE on transportation of triglycerides, cholesterol, and PC, we added native ApoE or ox-ApoE in cultured THP-1 foam cells. Ox-ApoE decreased function of THP-1 cells in efflux of triglycerides, cholesterol, and PC, comparing with native ApoE (Fig. 5A, B, and C). Consistently, less triglycerides, cholesterol, and PC in supernatant were detected in ox-ApoE groups than that of the native ApoE group (Fig. 5D, E, and F). Our data suggest that the oxidization of ApoE impairs its function of lipid transportation from foam cells.

### Ox-ApoE Exhibits Less Function in Clearing Plasma Triglyceride

**Figure 6:**
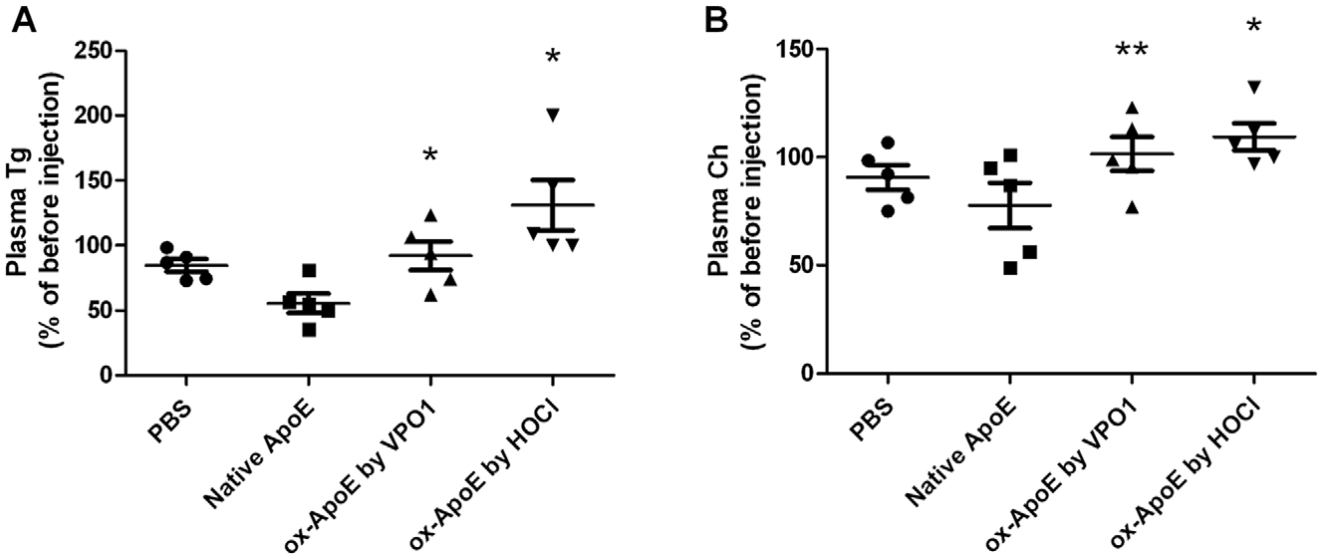
Effect of ox-ApoE on the transportation of plasma triglycerides and cholesterol. A. Changes of plasma triglycerides by administration of native and ox-ApoE. B. Changes of plasma cholesterol by administration of native and ox-ApoE. Plasma triglyceride and cholesterol concentrations were measured 1 hr before and after of ApoE administration. *p<0.05 *vs*. native ApoE. **p<0.05 *vs*. native ApoE (paired t-test). n = 5.

Previous studies on mice with transgenic expression of ApoE and mice transfected with virus containing ApoE indicated that ApoE facilitated plasma lipid clearance by liver [10], [11], [12]. In the present study, we ask the effect of ox-ApoE oxidized by VPO1 on transportation and clearance of triglyceride in plasma. We injected PBS, native ApoE or ox-ApoE (either by VPO1 or reagent HOCl) into the circulation *via* mouse tail vein. To minimize effects of the stress on triglyceride levels, plasma was taken 1 hr before injection. After 1 hr of injection, plasma was taken for analysis. The concentrations of triglycerides were measured. Triglyceride concentrations at pre- and post-injection were compared and the percentage changes were expressed. Decreased plasma triglyceride concentrations were observed after native ApoE administration, comparing with PBS groups (Fig. 6A). Significantly, ox-ApoE increased plasma triglyceride levels. Both reagent HOCl-oxidized ApoE and VPO1-oxidized ApoE had the similar effects (Fig. 6A). Similarly, plasma cholesterol concentrations were decreased by administrating native ApoE whereas slight increase was observed by administrating ox-ApoE (Fig. 6B).

## Discussion

The milestone of genesis of atherosclerosis is the deposition of lipoprotein in vessel walls [40]. ApoE is an important protein component of triglyceride-rich lipoprotein complexes including chylomicron, VLDL, IDL, and HDL. These complexes play a crucial role in transportation, distribution and catabolism of triglyceride-rich lipoproteins in plasma. Increased levels of ApoE facilitate lipid transportation and catabolism whereas decreased levels of ApoE lead to less transportation of lipids, resulting in elevated triglyceride levels in plasma and lipid deposition in vessel walls [10], [11], [12]. ApoE accomplishes its lipid transport and delivery function mainly through VLDL receptor. Therefore, abnormal expression or structural modifications of ApoE will cause its dysfunction. For example, ApoE-deficient mice spontaneously develop atherosclerotic lesions on a standard chow diet [41]. Ox-ApoE oxidized by MPO has impaired function for transporting lipids [32]. Our previous report demonstrated that VPO1 is the second member of hPx family generating potent oxidant, HOCl [20]. In the present study, we further define that VPO1 can, like MPO or reagent HOCl, oxidize ApoE, and impair the ability of ApoE to transport lipids, causing elevated plasma lipid levels.

Oxidative modification of ApoE leads to its structure changes and reduced transportation of lipids. The major lipid-binding domain of ApoE is at the C-terminal (amino acids 241–272) [42]. This region shows higher hydrophobicity on the surface. The present study reveals that amino acid residues of ApoE are widely oxidized, especially that of the hydroxyl group, carboxyl group, and imidazole group. The modification of these residues causes changes of the lipid-binding capacity of ApoE in two ways. One is the damage of its lipid binding domain, causing less lipid attachment [43]. Another is the oxidation of surface polar residues, turning ApoE more hydrophobic [44]. Our data show that the latter seems dominant. The ability of ox-ApoE for the transportation of lipids into cells is impaired, assuming that ox-ApoE has decreased ability to bind to VLDL receptor. Furthermore, ox-ApoE becomes unstable and prone to deposit in the vessel walls. In addition to oxidize ApoE, VPO1 may also oxidize other lipid proteins such as apolipoprotein B, apolipoprotein A-I, apolipoprotein A-II in a similar way since the potent oxidants generated by VPO1 oxidize a variety of proteins without selectivity.

Unlike MPO, which restrictedly expresses in monocytes and neutrophils, VPO1 highly expresses in a variety of tissues including cardiovascular system, lung and liver and is secreted into blood [18]. The plasma VPO1 concentration is approximately three orders of magnitude of that of MPO [19]. Interestingly, VPO1 and ApoE are co-located at the plaque of atherosclerotic aorta from LDLR −/− mice. These data implicate that VPO1 participates ApoE metabolism in plasma as well as in vessel walls at inflammatory sites.

It is well known that inflammatory factors play a key role in genesis and development of atherosclerosis. Lipopolysaccharides (LPS) induce the migration and activation of neutrophils [45]. Ox-LDL stimulates macrophages to release a variety of inflammatory factors including TNF-α and mediates migration and proliferation of smooth muscle cells [46], [47], [48]. The interplay of oxidized lipids and inflammatory factors improve the progress of atherosclerosis. MPO is currently considered as one of major mediators in the genesis and development of atherosclerosis. Like MPO, VPO1 expression is also regulated by inflammatory factors such as TNF-α and LPS [19]. VPO1 is located at the inflammatory sites in vessel walls. Thus, the inflammatory factors and increased expression of VPO1 may interplay in genesis and development of atherosclerosis. We propose that VPO1 is a new and important mediator for genesis and development of atherosclerosis. One question is which one plays a major role in genesis of atherosclerosis? Because MPO is distributed in inflammatory cells with approximately 5% of dry weight of the cell and has stronger peroxidase activity, we hypothesize that MPO may play a dominant role in the acute stage of atherosclerosis. However, the basal level of VPO1 is relative higher and it is also induced by a variety of inflammatory factors as our previous report that LPS and TNF-α induced VPO1 expression and secretion in HUVECs [19]. Indeed, increased levels of VPO1 were seen in the stable angina pectoris and acute coronary symptom (Unpublished observation). Thus, it is currently unclear if VPO1 plays a major role in atherosclerosis. It is important, but beyond our present study.

H_2_O_2_ is the co-substrate of VPO1. The major sources of H_2_O_2_ in vessel walls include mitochondrial electron transport chain, NADPH oxidases (Noxes) and uncoupled nitric oxide synthase [49], [50]. Noxes are professional enzymes for generation of reactive oxygen species and are considered to be regulated deliberately [25]. Former studies had proved that there were several Nox genes expressed in epithelial cells and atherosclerotic plaques [51], [52]. Of interest, at the inflammatory sites, a number of chemokines and cytokines induce the expression and activation of Noxes. For example, TNF-α induced Nox2 activation *via* PKC-mediated phosphorylation of p47phox in endothelial cells [53]. In addition, it is well known that hPxes are functionally associated with Noxes. MPO utilizes Nox2-generated H_2_O_2_ (derived from superoxide by dismutation) to produce HOCl in host defense. Pathologically, synergic actions of MPO and Noxes produce HOCl and oxidize ApoE [18]. We previously found that VPO1 may regulate the proliferation of vascular smooth muscle cells *via* Nox/H_2_O_2_/VPO1/HOCl/ERK1/2 pathway and involve in ox-LDL-induced endothelial cell apoptosis through Nox/p38 MAPK/caspase-3 pathway [54], [55]. Thus, we hypothesize that Nox-derived H_2_O_2_ is the major source of VPO1 catalysis in vessel walls and plasma.

### Conclusions

In the present study, we investigated the role of VPO1 in regulating ApoE-mediated lipid metabolism and transportation. We found that ApoE and VPO1 are co-localized at atherosclerotic plaques. VPO1-mediated oxidation of ApoE causes decreased efflux and impaired clearance of plasma lipids. We suggest that VPO1 is a new mediator of genesis and development of atherosclerosis.

## References

[1] Choy PC, Siow YL, Mymin D, Karmin O (2004) Lipids and atherosclerosis. Biochem Cell Biol 82: 212–224. 15052339 10.1139/o03-085

[2] Cox RA, Garcia-Palmieri MR (1990) Cholesterol, Triglycerides, and Associated Lipoproteins. In: Walker HK, Hall WD, Hurst JW, editors. Clinical Methods: The History, Physical, and Laboratory Examinations. 3rd ed. Boston.

[3] Mahley RW, Innerarity TL, Rall SC Jr, Weisgraber KH (1984) Plasma lipoproteins: apolipoprotein structure and function. J Lipid Res 25: 1277–1294. 6099394

[4] Mahley RW (1988) Apolipoprotein E: cholesterol transport protein with expanding role in cell biology. Science 240: 622–630. 3283935 10.1126/science.3283935

[5] Sing CF, Davignon J (1985) Role of the apolipoprotein E polymorphism in determining normal plasma lipid and lipoprotein variation. Am J Hum Genet 37: 268–285. 3985008 PMC1684560

[6] Plump AS, Smith JD, Hayek T, Aalto-Setala K, Walsh A, et al (1992) Severe hypercholesterolemia and atherosclerosis in apolipoprotein E-deficient mice created by homologous recombination in ES cells. Cell 71: 343–353. 1423598 10.1016/0092-8674(92)90362-g

[7] Zhang SH, Reddick RL, Piedrahita JA, Maeda N (1992) Spontaneous hypercholesterolemia and arterial lesions in mice lacking apolipoprotein E. Science. 258: 468–471. 10.1126/science.1411543 1411543

[8] Nakashima Y, Plump AS, Raines EW, Breslow JL, Ross R (1994) ApoE-deficient mice develop lesions of all phases of atherosclerosis throughout the arterial tree. Arterioscler Thromb 14: 133–140. 8274468 10.1161/01.atv.14.1.133

[9] Reddick RL, Zhang SH, Maeda N (1994) Atherosclerosis in mice lacking apo E. Evaluation of lesional development and progression. Arterioscler Thromb 14: 141–147. 8274470 10.1161/01.atv.14.1.141

[10] Shimano H, Ohsuga J, Shimada M, Namba Y, Gotoda T, et al (1995) Inhibition of diet-induced atheroma formation in transgenic mice expressing apolipoprotein E in the arterial wall. J Clin Invest 95: 469–476. 7860728 10.1172/JCI117687 PMC295491

[11] Hasty AH, Linton MF, Brandt SJ, Babaev VR, Gleaves LA, et al (1999) Retroviral gene therapy in ApoE-deficient mice: ApoE expression in the artery wall reduces early foam cell lesion formation. Circulation 99: 2571–2576. 10330390 10.1161/01.cir.99.19.2571

[12] Gaudreault N, Kumar N, Olivas VR, Eberle D, Rapp JH, et al (2012) Macrophage-specific apoE gene repair reduces diet-induced hyperlipidemia and atherosclerosis in hypomorphic Apoe mice. PLoS One 7: e35816. 22606237 10.1371/journal.pone.0035816 PMC3351426

[13] Steinberg D, Parthasarathy S, Carew TE, Khoo JC, Witztum JL (1989) Beyond cholesterol. Modifications of low-density lipoprotein that increase its atherogenicity. N Engl J Med 320: 915–924. 2648148 10.1056/NEJM198904063201407

[14] Luc G, Fruchart JC (1991) Oxidation of lipoproteins and atherosclerosis. Am J Clin Nutr 53: 206S–209S. 1985389 10.1093/ajcn/53.1.206S

[15] Park JG, Oh GT (2011) The role of peroxidases in the pathogenesis of atherosclerosis. BMB Rep 44: 497–505. 21871172 10.5483/bmbrep.2011.44.8.497

[16] Podrez EA, Abu-Soud HM, Hazen SL (2000) Myeloperoxidase-generated oxidants and atherosclerosis. Free Radic Biol Med 28: 1717–1725. 10946213 10.1016/s0891-5849(00)00229-x

[17] Sugiyama S, Okada Y, Sukhova GK, Virmani R, Heinecke JW, et al (2001) Macrophage myeloperoxidase regulation by granulocyte macrophage colony-stimulating factor in human atherosclerosis and implications in acute coronary syndromes. Am J Pathol 158: 879–891. 11238037 10.1016/S0002-9440(10)64036-9 PMC1850342

[18] Cheng G, Salerno JC, Cao Z, Pagano PJ, Lambeth JD (2008) Identification and characterization of VPO1, a new animal heme-containing peroxidase. Free Radic Biol Med 45: 1682–1694. 18929642 10.1016/j.freeradbiomed.2008.09.009 PMC2659527

[19] Cheng G, Li H, Cao Z, Qiu X, McCormick S, et al (2011) Vascular peroxidase-1 is rapidly secreted, circulates in plasma, and supports dityrosine cross-linking reactions. Free Radic Biol Med 51: 1445–1453. 21798344 10.1016/j.freeradbiomed.2011.07.002 PMC3439998

[20] Li H, Cao Z, Moore DR, Jackson PL, Barnes S, et al (2012) Microbicidal Activity of Vascular Peroxidase 1 in Human Plasma via Generation of Hypochlorous Acid. Infect Immun 80: 2528–2537. 22526679 10.1128/IAI.06337-11 PMC3416459

[21] Li H, Cao Z, Zhang G, Thannickal VJ, Cheng G (2012) Vascular peroxidase 1 catalyzes the formation of hypohalous acids: characterization of its substrate specificity and enzymatic properties. Free Radic Biol Med 53: 1954–1959. 22982576 10.1016/j.freeradbiomed.2012.08.597 PMC3506185

[22] Havel RJ, Eder HA, Bragdon JH (1955) The distribution and chemical composition of ultracentrifugally separated lipoproteins in human serum. J Clin Invest 34: 1345–1353. 13252080 10.1172/JCI103182 PMC438705

[23] Chantepie S, Malle E, Sattler W, Chapman MJ, Kontush A (2009) Distinct HDL subclasses present similar intrinsic susceptibility to oxidation by HOCl. Arch Biochem Biophys 487: 28–35. 19464255 10.1016/j.abb.2009.05.005 PMC3070237

[24] Hazell LJ, Stocker R (1993) Oxidation of low-density lipoprotein with hypochlorite causes transformation of the lipoprotein into a high-uptake form for macrophages. Biochem J 290 (Pt 1): 165–172. 10.1042/bj2900165 PMC1132397 8439285

[25] Lambeth JD (2004) NOX enzymes and the biology of reactive oxygen. Nat Rev Immunol 4: 181–189. 15039755 10.1038/nri1312

[26] Tian L, Luo N, Klein RL, Chung BH, Garvey WT, et al (2009) Adiponectin reduces lipid accumulation in macrophage foam cells. Atherosclerosis 202: 152–161. 18511057 10.1016/j.atherosclerosis.2008.04.011 PMC2630479

[27] Hasty AH, Linton MF, Swift LL, Fazio S (1999) Determination of the lower threshold of apolipoprotein E resulting in remnant lipoprotein clearance. J Lipid Res 40: 1529–1538. 10428991

[28] Babaev VR, Dergunov AD, Chenchik AA, Tararak EM, Yanushevskaya EV, et al (1990) Localization of apolipoprotein E in normal and atherosclerotic human aorta. Atherosclerosis 85: 239–247. 2102087 10.1016/0021-9150(90)90116-z

[29] O'Brien KD, Deeb SS, Ferguson M, McDonald TO, Allen MD, et al (1994) Apolipoprotein E localization in human coronary atherosclerotic plaques by in situ hybridization and immunohistochemistry and comparison with lipoprotein lipase. Am J Pathol 144: 538–548. 8129039 PMC1887086

[30] Lassegue B, Griendling KK (2010) NADPH oxidases: functions and pathologies in the vasculature. Arterioscler Thromb Vasc Biol 30: 653–661. 19910640 10.1161/ATVBAHA.108.181610 PMC2841695

[31] Jolivalt C, Leininger-Muller B, Drozdz R, Naskalski JW, Siest G (1996) Apolipoprotein E is highly susceptible to oxidation by myeloperoxidase, an enzyme present in the brain. Neurosci Lett 210: 61–64. 8762192 10.1016/0304-3940(96)12661-6

[32] Jolivalt C, Leininger-Muller B, Bertrand P, Herber R, Christen Y, et al (2000) Differential oxidation of apolipoprotein E isoforms and interaction with phospholipids. Free Radic Biol Med 28: 129–140. 10656299 10.1016/s0891-5849(99)00232-4

[33] Panzenboeck U, Raitmayer S, Reicher H, Lindner H, Glatter O, et al (1997) Effects of reagent and enzymatically generated hypochlorite on physicochemical and metabolic properties of high density lipoproteins. J Biol Chem 272: 29711–29720. 9368040 10.1074/jbc.272.47.29711

[34] Marsche G, Hammer A, Oskolkova O, Kozarsky KF, Sattler W, et al (2002) Hypochlorite-modified high density lipoprotein, a high affinity ligand to scavenger receptor class B, type I, impairs high density lipoprotein-dependent selective lipid uptake and reverse cholesterol transport. J Biol Chem 277: 32172–32179. 12070141 10.1074/jbc.M200503200

[35] Malle E, Marsche G, Panzenboeck U, Sattler W (2006) Myeloperoxidase-mediated oxidation of high-density lipoproteins: fingerprints of newly recognized potential proatherogenic lipoproteins. Arch Biochem Biophys 445: 245–255. 16171772 10.1016/j.abb.2005.08.008

[36] Jerlich A, Hammel M, Nigon F, Chapman MJ, Schaur RJ (2000) Kinetics of tryptophan oxidation in plasma lipoproteins by myeloperoxidase-generated HOCl. Eur J Biochem 267: 4137–4143. 10866816 10.1046/j.1432-1327.2000.01449.x

[37] Takahashi S, Suzuki J, Kohno M, Oida K, Tamai T, et al (1995) Enhancement of the binding of triglyceride-rich lipoproteins to the very low density lipoprotein receptor by apolipoprotein E and lipoprotein lipase. J Biol Chem 270: 15747–15754. 7797576 10.1074/jbc.270.26.15747

[38] Lin CY, Duan H, Mazzone T (1999) Apolipoprotein E-dependent cholesterol efflux from macrophages: kinetic study and divergent mechanisms for endogenous versus exogenous apolipoprotein E. J Lipid Res. 40: 1618–1627. 10484608

[39] Smith JD, Miyata M, Ginsberg M, Grigaux C, Shmookler E, et al (1996) Cyclic AMP induces apolipoprotein E binding activity and promotes cholesterol efflux from a macrophage cell line to apolipoprotein acceptors. J Biol Chem 271: 30647–30655. 8940040 10.1074/jbc.271.48.30647

[40] Singh U, Jialal I (2006) Oxidative stress and atherosclerosis. Pathophysiology 13: 129–142. 16757157 10.1016/j.pathophys.2006.05.002

[41] Meir KS, Leitersdorf E (2004) Atherosclerosis in the apolipoprotein-E-deficient mouse: a decade of progress. Arterioscler Thromb Vasc Biol 24: 1006–1014. 15087308 10.1161/01.ATV.0000128849.12617.f4

[42] Chang S, ran Ma T, Miranda RD, Balestra ME, Mahley RW, et al (2005) Lipid- and receptor-binding regions of apolipoprotein E4 fragments act in concert to cause mitochondrial dysfunction and neurotoxicity. Proc Natl Acad Sci U S A 102: 18694–18699. 16344479 10.1073/pnas.0508254102 PMC1311737

[43] Saito H, Dhanasekaran P, Baldwin F, Weisgraber KH, Lund-Katz S, et al (2001) Lipid binding-induced conformational change in human apolipoprotein E. Evidence for two lipid-bound states on spherical particles. J Biol Chem 276: 40949–40954. 11533033 10.1074/jbc.M106337200

[44] Sharp JS, Tomer KB (2007) Analysis of the oxidative damage-induced conformational changes of apo- and holocalmodulin by dose-dependent protein oxidative surface mapping. Biophys J 92: 1682–1692. 17158574 10.1529/biophysj.106.099093 PMC1796823

[45] Dornelles FN, Santos DS, Van Dyke TE, Calixto JB, Batista EL Jr, et al (2009) In vivo up-regulation of kinin B1 receptors after treatment with Porphyromonas gingivalis lipopolysaccharide in rat paw. J Pharmacol Exp Ther 330: 756–763. 19561153 10.1124/jpet.109.155762

[46] Jovinge S, Ares MP, Kallin B, Nilsson J (1996) Human monocytes/macrophages release TNF-alpha in response to Ox-LDL. Arterioscler Thromb Vasc Biol 16: 1573–1579. 8977464 10.1161/01.atv.16.12.1573

[47] Itabe H, Obama T, Kato R (2011) The Dynamics of Oxidized LDL during Atherogenesis. J Lipids 2011: 418313. 21660303 10.1155/2011/418313 PMC3108093

[48] Kalayoglu MV, Hoerneman B, LaVerda D, Morrison SG, Morrison RP, et al (1999) Cellular oxidation of low-density lipoprotein by Chlamydia pneumoniae. J Infect Dis 180: 780–790. 10438367 10.1086/314931

[49] Pennathur S, Heinecke JW (2007) Oxidative stress and endothelial dysfunction in vascular disease. Curr Diab Rep 7: 257–264. 17686400 10.1007/s11892-007-0041-3

[50] Szasz T, Thakali K, Fink GD, Watts SW (2007) A comparison of arteries and veins in oxidative stress: producers, destroyers, function, and disease. Exp Biol Med (Maywood) 232: 27–37. 17202583

[51] Guzik TJ, West NE, Black E, McDonald D, Ratnatunga C, et al (2000) Vascular superoxide production by NAD(P)H oxidase: association with endothelial dysfunction and clinical risk factors. Circ Res 86: E85–90. 10807876 10.1161/01.res.86.9.e85

[52] Guzik TJ, Chen W, Gongora MC, Guzik B, Lob HE, et al (2008) Calcium-dependent NOX5 nicotinamide adenine dinucleotide phosphate oxidase contributes to vascular oxidative stress in human coronary artery disease. J Am Coll Cardiol 52: 1803–1809. 19022160 10.1016/j.jacc.2008.07.063 PMC2593790

[53] Frey RS, Rahman A, Kefer JC, Minshall RD, Malik AB (2002) PKCzeta regulates TNF-alpha-induced activation of NADPH oxidase in endothelial cells. Circ Res 90: 1012–1019. 12016268 10.1161/01.res.0000017631.28815.8e

[54] Shi R, Hu C, Yuan Q, Yang T, Peng J, et al (2011) Involvement of vascular peroxidase 1 in angiotensin II-induced vascular smooth muscle cell proliferation. Cardiovasc Res 91: 27–36. 21292788 10.1093/cvr/cvr042 PMC3112017

[55] Bai YP, Hu CP, Yuan Q, Peng J, Shi RZ, et al (2011) Role of VPO1, a newly identified heme-containing peroxidase, in ox-LDL induced endothelial cell apoptosis. Free Radic Biol Med 51: 1492–1500. 21820048 10.1016/j.freeradbiomed.2011.07.004 PMC3570029

